# Long term circulation of avian paramyxovirus 4 in Australia revealed by historical and contemporary genomes from wild and domestic birds

**DOI:** 10.1186/s12985-026-03211-4

**Published:** 2026-06-22

**Authors:** Michelle Wille, Vittoria Stevens, Sebastian V. Carmody, Madeline Belfrage, Mia Campling, Suzanne L. Lowther, Kelly Davies, James O’Dwyer, Sue Martin, Marcel Klaassen, John S. Mackenzie, Matthew J. Neave, Jasmina M. Luczo

**Affiliations:** 1 WHO Collaborating Centre for Reference and Research on Influenza, Peter Doherty Institute for Infection and Immunity, Melbourne, 3000 VIC Australia; 2 Department of Microbiology and Immunology, University of Melbourne, Peter Doherty Institute for Infection and Immunity, Melbourne, 3000 VIC Australia; 3 Australian Centre for Disease Preparedness, Commonwealth Scientific and Industrial Research Organisation, East Geelong, 3219 VIC Australia; 4 Department of Natural Resources and Environment, Prospect, 7000 TAS Australia; 5 School of Life and Environmental Sciences, Deakin University, Geelong, 3217 VIC Australia; 6 Faculty of Health Sciences, Curtin University, Bentley, 6102 WA Australia

**Keywords:** Australia, Avian paramyxovirus 4, APMV4, Disease ecology, Wild birds

## Abstract

Avian paramyxovirus 4 (APMV4) is detected sporadically in wild birds and poultry, globally. While predominantly detected in waterfowl, the natural reservoir and ecology of APMV4 remains unclear. Herein we report historical and contemporary detections of APMV4 in Australia, and through sequencing demonstrate the likely long-term presence in a diversity of Australian wild bird species. This is evidenced by contemporary Australian genome sequences being more similar to historical Australian genomes rather than contemporary Asian genomes and that these genomes form a single lineage. This suggests that APMV4 has been circulating on the continent for decades. While APMV4 has demonstrable, albeit sporadic, impact on poultry globally, there has never been a report of this virus causing disease in poultry in Australia, suggesting it likely continues to pose a low risk for the poultry industry. As wild birds serve as natural reservoirs for numerous viruses of potential concern to poultry, dedicated surveillance is critical for revealing the risk profile of wild bird viruses, such as APMV4, to poultry.

**Supplementary Information:**

The online version contains supplementary material available at 10.1186/s12985-026-03211-4.

## Introduction

Avian paramyxoviruses (Family *Paramyxoviridae*, subfamily *Avulavirinae*) are important pathogens of domestic birds. There are 28 virus species described across three genera (September 2025) [1]. To date, global surveillance and characterisation efforts have focused on avian paramyxovirus 1 (*Orthoavulavirus javaense*), the causative agent of Newcastle disease, a severe poultry disease causing significant animal welfare and socioeconomic impacts [2–6]. In addition to velogenic (high virulence) and mesogenic (moderate virulence) strains that cause disease in poultry and pigeons and have also been detected in wild birds [7, 8], a diversity of lentogenic (low virulence) strains circulate in wild birds, which are considered to be the natural reservoirs [5, 6, 9–11]. Of the remaining 22 virus species, most have no significant economic impact in poultry, but some (e.g. avian paramyxovirus 2, 3, 4, 6 and 7) may cause respiratory diseases and reproductive problems in turkeys, ostriches, and chickens [5, 12].

Avian paramyxovirus 4 (APMV4) (*Paraavulavirus hongkongense*) was first identified in 1975 in ducks in Hong Kong [13, 14]. In chickens, APMV4 has been associated with an increase in white-shelled eggs, mild interstitial pneumonia, and catarrhal tracheitis [5, 12, 15]. Globally, beyond poultry, it has been detected in a diversity of wild bird species and notably waterfowl [16–21]. Wild waterfowl are suspected to be the natural reservoir for these viruses, however beyond sporadic detections, the ecology of APMV4 in the wild bird reservoir is unknown and the transmission dynamics between wild birds and poultry remains unresolved. Given its potential to cause disease in poultry and high likelihood that wild birds play a role as reservoirs for this virus, we aimed to clarify the status of APMV4 in Australia. Whilst there has been two historical reports of APMV4 in Australian wild birds [21, 22], this virus has never been detected through any targeted APMV surveillance activities and there are no sequences available from Australian birds. To address this, we retrieved historical isolates (from 1980 to 1994) from the Australian Centre for Disease Preparedness virus repository including samples obtained through the Wildlife Exotic Disease Preparedness Program (WEDPP). We also included opportunistic detections from contemporary samples (since 2017) collected for avian influenza and avian paramyxovirus 1 surveillance, as part of a diversity of research programs, including the National Avian Influenza in Wild Birds (NAIWB) program. Herein we demonstrate that not only is APMV4 present in Australia, its likely to have been circulating in Australia among a diversity of wild bird species for decades. This study highlights the limited current understanding of avian paramyxoviruses and that increased surveillance and investigation are likely to reveal greater diversity and prevalence than currently appreciated.

## Methods

### Historical samples

Details of sample collection in Western Australia and associated virus isolation is described in Mackenzie et al. (1984) [22, 23]. Briefly, between 1980 and 1981 cloacal swabs were collected from birds at three sites in Western Australia, placed in virus transport medium (VTM) and immediately frozen. Viruses were isolated in embryonated chicken eggs [22]. A historical Victorian wild duck sample was obtained as part of the Wildlife Exotic Disease Preparedness Program (WEDPP) [21]. Wild ducks were captured and swabs were collected into VTM, following which viruses were isolated in embryonated chicken eggs. Resulting isolates were archived at the Australian Centre for Disease Preparedness.

### Contemporary sample collection and screening

Currently, active surveillance is predominantly undertaken in Australia by the National Avian Influenza in Wild Birds Program (e.g [24]). for the purpose of avian influenza surveillance.

In Tasmania, faecal environmental swab samples were collected from a long-standing sampling site (see [25, 26]). The sample was initially extracted and screened for influenza A virus as per [25, 26] and avian paramyxovirus 1 using a combination of Taqman qPCR assays against the M, F, L genes [27, 28]. Virus isolation from swab material identified an agglutinating agent that underwent next generation sequencing.

In Victoria, combined oropharyngeal and cloacal samples were collected from live captured ducks for the predominant purpose of avian influenza surveillance. Details of sample collection and RNA extraction are presented in [29, 30]. Briefly, in June – July 2022, > 400 ducks were captured using canon-netting and walk in traps at 4 sites in Victoria, Australia. From each individual, a combined cloacal sample and an oropharyngeal swab were collected and placed into VTM. RNA was extracted using the NucleoMag VET Kit (Macherey-Nagel) on the Kingfisher Flex. RNA was screened for avian paramyxovirus 1 using a combination of Taqman qPCR assays against the M, F, L genes [27, 28]. Samples with a Ct < 32 were selected for virus isolation.

### Virus isolation

Virus isolation was performed via allantoic inoculation of 9-11-day-old specific pathogen free (SPF) embryonated chicken eggs and incubated for five days at 37 °C. Agglutinating agents were identified by haemagglutination assay of allantoic fluid using chicken erythrocytes. For contemporary samples, in cases where allantoic fluid was negative, they were passaged a second time. RNA was extracted from allantoic fluid using the MagMAX-96 viral RNA isolation kit (Thermo Fisher Scientific) on the Kingfisher Flex.

### Sequencing and bioinformatics

Dual-indexed DNA libraries were prepared using a total RNA stranded library preparation kit (Illumina). The quantity of the libraries were determined using a Qubit™ dsDNA High Sensitivity Assay kit (Thermofisher) and the quality verified using 2100 Bioanalyzer and Agilent High Sensitivity DNA kit (Integrated Sciences). Whole genome sequencing was undertaken on an Illumina NextSeq2000 instrument using a P1 reagent cartridge (2 × 150 bp paired end reads). On average 2–6 million reads were recovered per sample. Sequence reads were trimmed for quality and mapped to respective reference sequence using Geneious Prime software (www.geneious.com) (Biomatters, Auckland, NZ).

APMV4 genomes (partial and complete, *n* = 178) were downloaded from GenBank (14/05/2025), and further supplemented from genomes from An et al. (2024) [31]. We also queried GenBank using blast of both historical and contemporary fusion (F) genes and complete and coding complete genomes to ensure all available genomes were retrieved (14/05/2025). In all cases, sequences were aligned using MAFFT [32]. Maximum likelihood trees were constructed using IQ-Tree [33], integrating the most suitable nucleotide substitution model, and run with 1000 ultrafast bootstraps. We undertook time structured phylogenetic analysis using coding complete F genes. First, we performed a linear regression of root-to-tip distances against year of sampling with TempEst [34]. Subsequently, a time-scaled phylogenetic tree was estimated using BEAST v1.10.4 [35], under the uncorrelated lognormal relaxed clock [36] and SRD06 codon structured nucleotide substitution model [37], and the Bayesian skyline coalescent tree prior [38]. One hundred million generations were performed, and convergence was assessed using Tracer v1.8 (http://tree.bio.ed.ac.uk/software/tracer/). A maximum credibility lineage tree was generated using TreeAnnotator following the removal of 10% burn-in. All phylogenetic trees were visualised using Fig Tree v1.4 (http://tree.bio.ed.ac.uk/software/figtree/).

## Results

### First APMV4 genomes from Australia

**Fig. 1:**
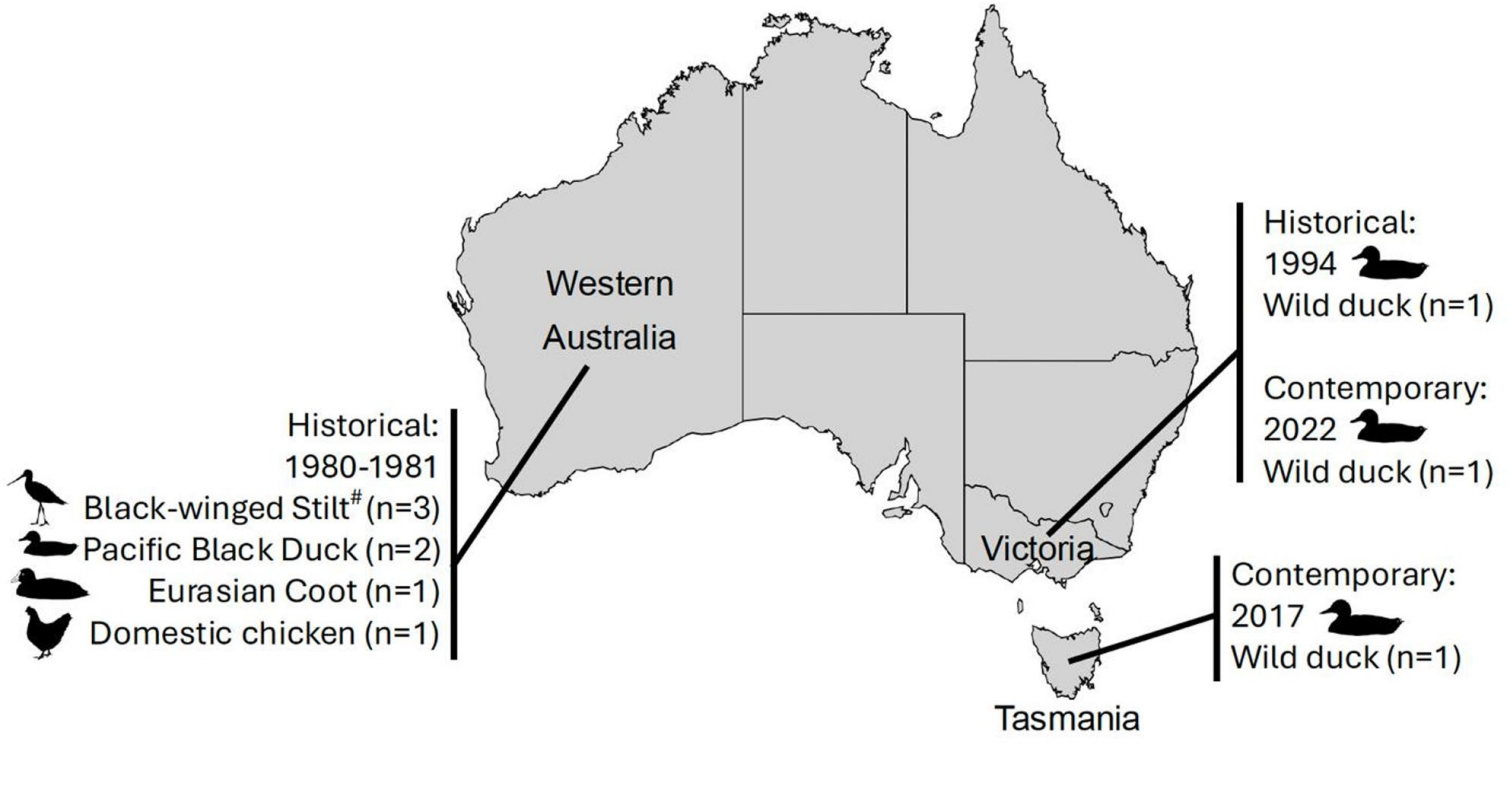
Map of Australia showing the reported locations (states) of where samples were collected. Silouttes from phylopic.org. # The original metadata associated with the samples lists “Black-winged Stilt” as the host, however the Australian sub-species of Black-winged Stilt (*Himantopus himantopus*) has been reclassified as Pied Stilt (*Himantopus leucocephalus*). As both names are still used, we have retained the species name as per the initial metadata

Despite not being the target of any active surveillance in Australia, we have sequenced ten coding complete APMV4 genomes from samples collected in Australia. Of these, eight comprise historical (1980–1994) isolates collected from four different species in Western Australia [22] and a wild duck in Victoria [21]. Contemporary isolates (since 2017) were those from samples collected by the NAIWB program and other surveillance systems in Tasmania and Victoria. Together, these ten coding complete genomes comprise the first APMV4 genomes sequenced from samples collected in Australia (Table 1; Fig. 1).

**Table 1:** APMV4 genomes sequenced in this study

Isolate	Host	State	Date	GenBank Accession
4341	Black-winged Stilt^#^ (*Himantopus himantopus*)	Western Australia	17/11/1980	PZ0243693
4427	Black-winged Stilt^#^ (*Himantopus himantopus*)	Western Australia	19/11/1980	PZ024394
4428	Black-winged Stilt^#^ (*Himantopus himantopus*)	Western Australia	19/11/1980	PZ024395
4958	Pacific Black Duck (*Anas superciliosa*)	Western Australia	05/03/1981	PZ024392
4967	Pacific Black Duck (*Anas superciliosa*)	Western Australia	05/03/1981	PZ024391
4988	Eurasian Coot (*Fulica atra*)	Western Australia	05/03/1981	PZ024397
5030	Domestic chicken (*Gallus gallus domesticus*)	Western Australia	27/03/1981	PZ024396
W192	Wild duck (*Anas sp.*)	Victoria	05/1994	PZ024399
17-0958-35	Wild duck (*Anas sp.*)	Tasmania	13/03/2017	PZ024400
15,805	Grey Teal (*Anas gracilis*)	Victoria	15/06/2022	PZ024398

# The original metadata associated with the samples lists “Black-winged Stilt” as the host, however the Australian sub-species of Black-winged Stilt (*Himantopus himantopus*) has been reclassified as Pied Stilt (*Himantopus leucocephalus*). As both names are still used, we have retained the species name as per the initial metadata

The generated genomes range from 15,005 bp (APMV4/Black Duck/Western Australia/4958/1981) to 15,047 bp (APMV4/Grey Teal/Victoria/15805/2022). Coverage statistics are available in Table S1. While we did recover all ORFs, all genomes had incomplete 3’ and 5’ UTRs (Fig S1). The deduced amino acid sequences of the F gene cleavage sites for all sequences generated in this study are ^116^DIQPR↓F^121^, which is consistent with other reported APMV4 viruses. While in avian paramyxovirus 1, the amino acid sequence of the F protein cleavage site has a relationship with pathogenicity, this relationship is less clear in other avian paramyxovirus species, including APMV4 [14]. There is no evidence of recombination in the genomes as blast analysis of each ORF consistently returns the same top hits for each virus strain (Table S2). Given the consistency of the blast results across ORFS, no further recombination analysis was undertaken.

Both blast and phylogenetic analysis indicate the most closely related viruses are from Asia. Overall, the mostly closely related sequences in GenBank, regardless of ORF include APMV4/duck/Hongkong/D3/75 (FJ177514), APMV4/northern pintail/Primorje/Russia/276/2019 (MW959125) and APMV4/Anseriformes/Taiwan/AHRI150/2019 (MZ802798) (Table S2).

### Sequences from Australia form a discrete clade

Given the availability of both historical and contemporary sequences, we aimed to understand whether there was a single clade of APMV4 circulating in Australia or whether there were multiple clades and substantial genetic diversity. To address this, we interrogated the genomes in context of available sequences at both the F gene level (as most sequences in Genbank comprise only the F gene) and the coding complete genome level.

Using percentage similarity calculations using the full F gene, the contemporary sequences generated in this study are more similar to the historical genomes we sequenced rather than sequences from GenBank. APMV4/wild duck/Victoria/W192/1994 is 96.1% similar to Australia genomes from 1980 to 1981 (which are 100% similar to each other), compared to 93.7% similar to APMV4/duck/Hongkong/D3/75 (FJ177514), 93.9% similar to APMV4/mallard/Minnesota/AI07-3119/2007 (KT732313) and related sequences. Similarly, APMV4/Grey Teal/Victoria/15,805/2022 is more similar to Australia genomes from 1980 to 1981 (92.2%) than to APMV4/duck/Hongkong/D3/75 (FJ177514, 90.4%) and other sequences in GenBank. This is consistent with the coding complete genomes.

**Fig. 2:**
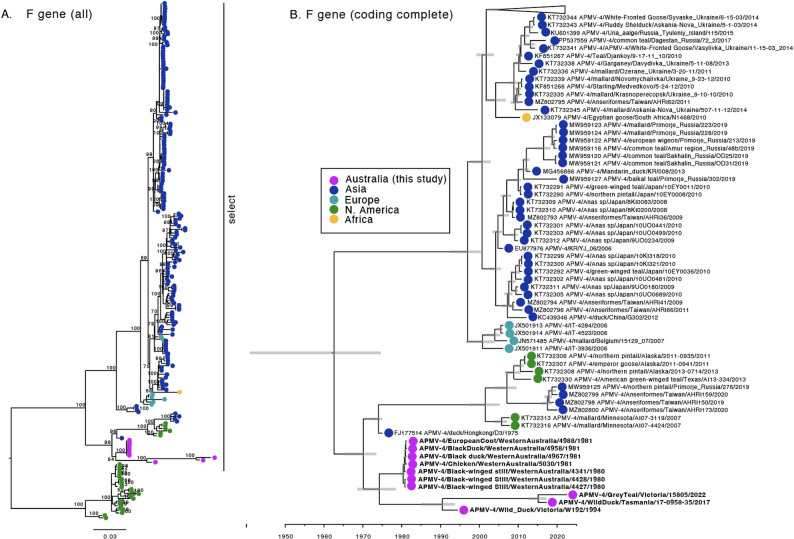
Phylogenetic tree of APMV4 F gene. Maximum likelihood tree of all (**A**) F gene sequences (partial > 250 bp and complete). Scale bar indicates the number of substitutions per site. Tree was midpoint rooted. (**B**) Time scaled phylogeny of coding complete F gene (1701 bp) from the clade comprising predominantly Eurasian and Australian sequences (i.e. clade indicated by “select” in A). The tree has been partially collapsed for clarity. Scale bar indicates years. Node bars represent the 95% HDP of node height. Tips are coloured by continent

Phylogenetic analysis of both partial (Fig. 2A) and coding complete F gene (Fig. 2B) and complete and coding complete genomes (Fig S2) indicate that all Australian sequences fall into a single clade. These sequences are sister to a clade comprising APMV4/duck/Hongkong/D3/75 (FJ177514) as well as more contemporary viruses from Alaska. The mean most recent common ancestor (MRCA) of the clade comprising the 10 sequences generated in this study is July 1974 (95% highest posterior density [HPD], December 1968 - February 1979) suggesting that this lineage arrived in Australia at least 50 years ago. Phylogenetic and blast results support long term, local circulation of a single lineage in Australia, although lack of publicly available sequences from 1980 to 2015 cause some uncertainty.

## Discussion

Herein we significantly advance the current understanding of APMV4 in Australia by demonstrating its long-term presence in a diversity of Australian wild and domestic bird species through sequencing of both historical and contemporary isolates.

The contemporary strains described in this study are not sister to those currently circulating in Asia. Rather, are most similar to historical strains from Australia and phylogenetic analysis of both the F gene and complete and coding complete genomes reveals all the Australian sequences fall into a single clade. The simplest explanation for this pattern is a single historical introduction of APMV4 into Australia, followed by prolonged, isolated circulation of this lineage over several decades.

Our findings align with existing knowledge of the evolutionary ecology of avian influenza viruses in Australia, where the continent can be considered a sink for viral diversity and introductions likely taking place through migratory shorebirds [26]. For example, certain subtypes such as H7 have been maintained in isolation within Australian wild bird populations, with sporadic jumps to poultry, since at least the 1970s. Conversely, other subtypes are subject to competitive exclusion following sporadic introductions of novel viral lineages from Asia or North America [26, 39]. A key limitation in revealing the evolutionary ecology of APMV4 is the paucity of global sequence data, particularly between 1980 and 2015. As such, while current evidence supports the hypothesis of long-term local persistence, the possibility of unobserved global diversity within this clade cannot be definitively ruled out.

While APMV4 has demonstrable impact on poultry [5, 12, 15], there have been no reports of APMV4-associated disease in Australian poultry to date. Given the evidence for long term circulation of APMV4 in Australian wild birds and the detection and isolation of APMV4 from backyard poultry, the absence of disease related outbreaks in commercial poultry suggests that APMV4 likely continues to pose a low risk for the poultry industry.

To date, the vast majority of APMV4 genomes, globally, are from samples collected from waterfowl. Indeed, all of our contemporary genomes are from waterfowl. However, whether waterfowl play a role as a reservoir for APMV4 is unclear, and the overrepresentation of waterfowl as hosts may be caused by sampling bias. Indeed, there is a strong focus on wild waterfowl for avian influenza surveillance, and often these same samples are used for surveillance of other avian viruses, including in Australia. Of note is that all the avian hosts from which we isolated APMVV4 are non-migratory, but are largely nomadic within the Australio-Papuan region, following water in the landscape, such that there is a inconsistency in patterns of where birds are in space and time [40–43]. That we did not detect APMV4 in migratory birds is more likely a reflection of biases in sampling and detection than ecology. In order to ascertain the ecology of this virus in wild birds, further eco-epidemiology studies are required, across a broader host range. Regardless, wild birds serve as natural reservoirs for numerous viruses of potential concern to poultry. It is likely that these viruses have co-evolved with their wild bird hosts, resulting in low or no virulence in wild bird populations. However, spillover events into domestic poultry may have significant consequences, as demonstrated by both avian influenza, but also the sporadic detections of avian paramyxovirus 2, 3, 4, 6, 7 in poultry [5, 12]. To improve risk assessments and preparedness for the poultry industry, it is essential to enhance our understanding of viral diversity and ecology in wild bird populations. This study underscores the value of catalogued historic repositories and longitudinal surveillance systems and flags the opportunity for discovery that is provided by integrated, multi-host, multi-pathogen surveillance systems that encompass both wild and domestic avian species.

### Ethics statement

As we retrieved archived virus isolates for historical samples, any animal ethics or permits would be under the purview of the original sample collectors [21, 23]. For contemporary isolates: faecal environmental sampling undertaken by the Department of Primary Industries, Parks, Water & Environment (Tasmania) does not require ethical approval. Capture and sampling of live ducks in Victoria was conducted under approval of Deakin University Animal Ethics Committee (application numbers B39-2019 and B28-2023), Wildlife Act 1975 Research Authorisation from the Victorian Department of Energy, Environment and Climate Action (permits 10009534, 10010870, and 10011005) and the Australian Bird and Bat Banding Scheme (banding authority 2915).

Contemporary work with embryonated eggs was reviewed and approved by the Australian Centre for Disease Preparedness Animal Ethics Committee (ACDP24005).

## Supplementary Information

Below is the link to the electronic supplementary material.

Supplementary Material 1.

## Data Availability

All genomes have been deposited in GenBank (Accession PZ024391- PZ024400; Table 1). Strain names follow the format of avian influenza designations for clarity. Trees are available on Github (https://github.com/michellewille2/APMV4).
