# Supplementary material for: Long term circulation of avian paramyxovirus 4 in Australia revealed by historical and contemporary genomes from wild and domestic birds
Source: Virol J. 2026 Jun 22;23:196. doi: 10.1186/s12985-026-03211-4 (PMC13474766; doi:10.1186/s12985-026-03211-4)
Supplement: Supplementary file 1 [file 12985_2026_3211_MOESM1_ESM.docx]

**Long term circulation of avian paramyxovirus 4 in Australia revealed by historical and contemporary genomes from wild and domestic birds.**

Michelle Wille, Vittoria Stevens, Sebastian V. Carmody, Madeline Belfrage, Mia Campling, Suzanne L. Lowther, Kelly Davies, James O’Dwyer, Sue Martin, Marcel Klaassen, John S. Mackenzie, Matthew J. Neave, Jasmina M. Luczo

**Supplemental Tables and Figures**

**Figure S1.** Genome arrangement of coding complete Australian APMV4 genomes.

**Figure S2.** Maximum likelihood of complete and near complete APMV4

**Table S1.** Coverage statistics for APMV-4 genomes sequenced in this study

**Table S2:** Top 3 blast hits for viruses sequenced in this study

**Figure S1.** Genome arrangement of coding complete Australian APMV4 genomes. Individual genes are shown as coloured rectangles containing the nucleotide length of each gene cds and the amino acid length of the encoded protein. The nucleotide length of the non-coding regions are indicated. Coding complete genome length are shown to the right.

**Figure S2.** Maximum likelihood of complete and coding complete (15071 bp) APMV4. Scale bar indicates the number of substitutions per site. Tree was midpoint rooted. Tips are coloured by continent.

**Table S1. Coverage statistics for APMV-4 genomes sequenced in this study**

| **Designation** | **Total Reads** | **Reads mapped** | **% mapped** | **Average depth** | **Min depth** | **Max depth** |
| --- | --- | --- | --- | --- | --- | --- |
| APMV4/Black-winged Stilt/Western Australia/4341/1980 | 2570812 | 1960291 | 76 | 18105 | 7 | 43657 |
| APMV4/Black-winged Stilt/Western Australia/4427/1980 | 2667258 | 1916840 | 72 | 17436 | 12 | 54246 |
| APMV4/Black-winged Stilt/Western Australia/4428/1980 | 3925162 | 1148388 | 29 | 9733 | 8 | 27910 |
| APMV4/Pacific Black Duck/Western Australia/4958/1981 | 167010 | 87399 | 52 | 801 | 81 | 2683 |
| APMV4/Pacific Black Duck/Western Australia/4967/1981 | 6712456 | 2351457 | 35 | 20878 | 15 | 49184 |
| APMV4/Eurasian coot/Western Australia/4988/1981 | 1897624 | 1353722 | 71 | 12844 | 7 | 32225 |
| APMV4/chicken/Western Australia/5030/1981 | 3713666 | 2925220 | 79 | 26680 | 11 | 64156 |
| APMV4/wild duck/Victoria/W192/1994 | 2603558 | 1288565 | 49 | 11629 | 16 | 32056 |
| APMV4/wild duck/Tasmania/17-0958-35/2017 | 6311714 | 5619425 | 89 | 53041 | 9 | 129558 |
| APMV4/Grey Teal/Victoria/15805/2022 | 5826034 | 2734072 | 47 | 24982 | 31 | 59122 |

**Table S1: Top 3 blast hits (29/10/2025) for viruses sequenced in this study**

**APMV4/Pacific Black Duck/Western Australia/4958/1981**

Note: All genomes from 1980 and 1981 are 100% identical**.**

| **ORF** | **Top blast hit** | **Top blast hit** | **% nt similarity** |
| --- | --- | --- | --- |
| N | FJ177514 | APMV4/duck/Hongkong/D3/75, complete genome | 98.00% |
|  | MZ802798 | APMV4/Anseriformes/Taiwan/AHRI150/2019, complete genome | 95.60% |
|  | MZ802800 | APMV4/Anseriformes/Taiwan/AHRI173/2020, complete genome | 95.50% |
| P | FJ177514 | APMV4/duck/Hongkong/D3/75, complete genome | 97.10% |
|  | MZ802800 | APMV4/Anseriformes/Taiwan/AHRI173/2020, complete genome | 93.80% |
|  | MW959125 | APMV4/northern pintail/Primorje/Russia/276/2019, complete genome | 93.70% |
| M | FJ177514 | APMV4/duck/Hongkong/D3/75, complete genome | 98.00% |
|  | MZ802798 | APMV4/Anseriformes/Taiwan/AHRI150/2019, complete genome | 93.90% |
|  | MW959125 | APMV4/northern pintail/Primorje/Russia/276/2019, complete genome | 93.80% |
| F | FJ177514 | APMV4/duck/Hongkong/D3/75, complete genome | 98.10% |
|  | KT732316 | APMV4/mallard/Minnesota/AI07-4424/2007 fusion protein gene, complete cds | 95.50% |
|  | KT732313 | APMV4/mallard/Minnesota/AI07-3119/2007 fusion protein gene, complete cds | 95.40% |
| HN | FJ177514 | APMV4/duck/Hongkong/D3/75, complete genome | 98.00% |
|  | D14031 | Avian paramyxovirus 4 gene for hemagglutinin-neuraminidase, complete cds | 98.00% |
|  | MW959125 | APMV4/northern pintail/Primorje/Russia/276/2019, complete genome | 94.40% |
| L | FJ177514 | APMV4/duck/Hongkong/D3/75, complete genome | 98.30% |
|  | MZ802798 | APMV4/Anseriformes/Taiwan/AHRI150/2019, complete genome | 95.00% |
|  | MW959125 | APMV4/northern pintail/Primorje/Russia/276/2019, complete genome | 95.00% |
| Full genome | FJ177514 | APMV4/duck/Hongkong/D3/75, complete genome | 97.90% |
|  | MZ802798 | APMV4/Anseriformes/Taiwan/AHRI150/2019, complete genome | 94.10% |
|  | MZ802799 | APMV4/Anseriformes/Taiwan/AHRI159/2020, complete genome | 94% |

**APMV4/Wild Duck/Victoria/W192/1994**

| **ORF** | **Top blast hit** | **Top blast hit** | **% nt similarity** |
| --- | --- | --- | --- |
| N | FJ177514 | APMV4/duck/Hongkong/D3/75, complete genome | 96.50% |
|  | MZ802798 | APMV4/Anseriformes/Taiwan/AHRI150/2019, complete genome | 93.60% |
|  | MZ802800 | APMV4/Anseriformes/Taiwan/AHRI173/2020, complete genome | 93.50% |
| P | FJ177514 | APMV4/duck/Hongkong/D3/75, complete genome | 95.30% |
|  | MW959125 | APMV4/northern pintail/Primorje/Russia/276/2019, complete genome | 92.60% |
|  | MZ802800 | APMV4/Anseriformes/Taiwan/AHRI173/2020, complete genome | 92.70% |
| M | FJ177514 | APMV4/duck/Hongkong/D3/75, complete genome | 96.20% |
|  | MW959125 | APMV4/northern pintail/Primorje/Russia/276/2019, complete genome | 92.70% |
|  | MZ802798 | APMV4/Anseriformes/Taiwan/AHRI150/2019, complete genome | 92.80% |
| F | FJ177514 | APMV4/duck/Hongkong/D3/75, complete genome | 96.10% |
|  | KT732316 | APMV4/mallard/Minnesota/AI07-4424/2007 fusion protein gene, complete cds | 93.70% |
|  | KT732313 | APMV4/mallard/Minnesota/AI07-3119/2007 fusion protein gene, complete cds | 93.90% |
| HN | D14031 | Avian paramyxovirus 4 gene for hemagglutinin-neuraminidase, complete cds | 96.70% |
|  | FJ177514 | APMV4/duck/Hongkong/D3/75, complete genome | 96.70% |
|  | MW959125 | APMV4/northern pintail/Primorje/Russia/276/2019, complete genome | 93.90% |
| L | FJ177514 | APMV4/duck/Hongkong/D3/75, complete genome | 96.10% |
|  | MW959125 | APMV4/northern pintail/Primorje/Russia/276/2019, complete genome | 93.10% |
|  | MZ802798 | APMV4/Anseriformes/Taiwan/AHRI150/2019, complete genome | 93.10% |
| Full genome | FJ177514 | APMV4/duck/Hongkong/D3/75, complete genome | 95.80% |
|  | MZ802798 | APMV4/Anseriformes/Taiwan/AHRI150/2019, complete genome | 92.30% |
|  | MZ802799 | APMV4/Anseriformes/Taiwan/AHRI159/2020, complete genome | 92% |

**APMV4/Wild Duck/Tasmania/17-0958-35/2017**

| **ORF** | **Top blast hit** | **Top blast hit** | **% nt similarity** |
| --- | --- | --- | --- |
| N | FJ177514 | APMV4/duck/Hongkong/D3/75, complete genome | 94.00% |
|  | MW959125 | APMV4/northern pintail/Primorje/Russia/276/2019, complete genome | 92.2 |
|  | MZ802798 | APMV4/Anseriformes/Taiwan/AHRI150/2019, complete genome | 92.30% |
| P | FJ177514 | APMV4/duck/Hongkong/D3/75, complete genome | 92.30% |
|  | MW959125 | APMV4/northern pintail/Primorje/Russia/276/2019, complete genome | 89.90% |
|  | MZ802800 | APMV4/Anseriformes/Taiwan/AHRI173/2020, complete genome | 90.00% |
| M | FJ177514 | APMV4/duck/Hongkong/D3/75, complete genome | 96.20% |
|  | MW959125 | APMV4/northern pintail/Primorje/Russia/276/2019, complete genome | 92.70% |
|  | MZ802798 | APMV4/Anseriformes/Taiwan/AHRI150/2019, complete genome | 92.80% |
| F | FJ177514 | APMV4/duck/Hongkong/D3/75, complete genome | 92.70% |
|  | KT732306 | APMV4/northern pintail/Alaska/2011-0935/2011 fusion protein gene, complete cds | 90.90% |
|  | KT732308 | APMV4/northern pintail/Alaska/2013-0714/2013 fusion protein gene, complete cds | 90.80% |
| HN | D14031 | Avian paramyxovirus 4 gene for hemagglutinin-neuraminidase, complete cds | 92.50% |
|  | FJ177514 | APMV4/duck/Hongkong/D3/75, complete genome | 92.00% |
|  | MW959125 | APMV4/northern pintail/Primorje/Russia/276/2019, complete genome | 89.70% |
| L | FJ177514 | APMV4/duck/Hongkong/D3/75, complete genome | 93.20% |
|  | MZ802798 | APMV4/Anseriformes/Taiwan/AHRI150/2019, complete genome | 90.80% |
|  | MZ802799 | APMV4/Anseriformes/Taiwan/AHRI159/2020, complete genome | 90.80% |
| Full genome | FJ177514 | APMV4/duck/Hongkong/D3/75, complete genome | 92.40% |
|  | MZ802798 | APMV4/Anseriformes/Taiwan/AHRI150/2019, complete genome | 89.70% |
|  | MZ802799 | APMV4/Anseriformes/Taiwan/AHRI159/2020, complete genome | 90% |

**APMV4/Grey Teal/Victoria/15805/2022**

| **ORF** | **Top blast hit** | **Top blast hit** | **% nt similarity** |
| --- | --- | --- | --- |
| N | FJ177514 | APMV4/duck/Hongkong/D3/75, complete genome | 93.40% |
|  | MW959125 | APMV4/northern pintail/Primorje/Russia/276/2019, complete genome | 91.8 |
|  | MZ802798 | APMV4/Anseriformes/Taiwan/AHRI150/2019, complete genome | 91.90% |
| P | FJ177514 | APMV4/duck/Hongkong/D3/75, complete genome | 91.50% |
|  | MW959125 | APMV4/northern pintail/Primorje/Russia/276/2019, complete genome | 89.50% |
|  | MZ802800 | APMV4/Anseriformes/Taiwan/AHRI173/2020, complete genome | 89.60% |
| M | FJ177514 | APMV4/duck/Hongkong/D3/75, complete genome | 94.10% |
|  | MW959125 | APMV4/northern pintail/Primorje/Russia/276/2019, complete genome | 90.80% |
|  | MZ802798 | APMV4/Anseriformes/Taiwan/AHRI150/2019, complete genome | 90.90% |
| F | FJ177514 | APMV4/duck/Hongkong/D3/75, complete genome | 92.40% |
|  | KT732306 | APMV4/northern pintail/Alaska/2011-0935/2011 fusion protein gene, complete cds | 90.70% |
|  | KT732308 | APMV4/northern pintail/Alaska/2013-0714/2013 fusion protein gene, complete cds | 90.60% |
| HN | D14031 | Avian paramyxovirus 4 gene for hemagglutinin-neuraminidase, complete cds | 92.60% |
|  | FJ177514 | APMV4/duck/Hongkong/D3/75, complete genome | 92.60% |
|  | MW959125 | APMV4/northern pintail/Primorje/Russia/276/2019, complete genome | 90.50% |
| L | FJ177514 | APMV4/duck/Hongkong/D3/75, complete genome | 92.70% |
|  | MZ802798 | APMV4/Anseriformes/Taiwan/AHRI150/2019, complete genome | 90.40% |
|  | MZ802799 | APMV4/Anseriformes/Taiwan/AHRI159/2020, complete genome | 90.30% |
| Full genome | FJ177514 | APMV4/duck/Hongkong/D3/75, complete genome | 91.90% |
|  | MZ802798 | APMV4/Anseriformes/Taiwan/AHRI150/2019, complete genome | 89.30% |
|  | MZ802799 | APMV4/Anseriformes/Taiwan/AHRI159/2020, complete genome | 89% |
